# Radiobiological evaluation of hypofractionated vs. conventional VMAT for synchronous bilateral breast cancer: a focus on regional nodal irradiation

**DOI:** 10.3389/fmed.2026.1909316

**Published:** 2026-09-30

**Authors:** Jiyong Zhang, Danli Hong, Chunyan Deng

**Affiliations:** 1 Department of Radiation Oncology, Guangdong Engineering Research Center of AI-Powered Precision Cancer Diagnostics and Therapeutics (Proposed)/GuangDong Engineering Technology Research Center of AI-Powered Precision Cancer Diagnostics and Therapeutics, Cancer Hospital of Shantou University Medical College, Shantou, Guangdong, China; 2 Department of Endocrinology, The First Affiliated Hospital of Shantou University Medical College, Shantou, Guangdong, China

**Keywords:** hypofractionated radiotherapy, normal tissue complication probability, regional nodal irradiation, synchronous bilateral breast cancer, tumor control probability, volumetric modulated arc therapy

## Abstract

**Purpose:**

To compare the radiobiological characteristics of hypofractionated radiotherapy (HFRT) and conventional fractionated radiotherapy (CFRT) delivered with volumetric modulated arc therapy (VMAT) for synchronous bilateral breast cancer (SBBC) requiring regional nodal irradiation (RNI).

**Methods:**

Eighteen patients with SBBC receiving bilateral breast/chest wall irradiation and comprehensive nodal coverage were retrospectively included. For each patient, VMAT plans were generated for CFRT (50 Gy in 25 fractions) and HFRT (40.05 Gy in 15 fractions). Dosimetric parameters of the planning target volume (PTV) were evaluated. Radiobiological assessment included equivalent uniform dose (EUD), tumor control probability (TCP), and normal tissue complication probability (NTCP). TCP was calculated using LQ-Poisson and Niemierko models, while NTCP for the lungs, heart, spinal cord, and esophagus was estimated using Lyman Kutcher Burman and Niemierko models.

**Results:**

Both regimens achieved excellent target coverage, with mean PTV V_95%_ values exceeding 99%. HFRT significantly reduced high-dose regions within the PTV, as reflected by lower V_107%_ values (median: 1.35% vs. 2.60%; IQR: 5.75% vs. 7.85%; *P* < 0.001). PTV EUD was significantly lower with HFRT than with CFRT (median: 45.69 vs. 51.34 Gy; *P* < 0.001). Although TCP values were lower with HFRT in both models, tumor control remained high for both schedules. HFRT also significantly reduced EUD and NTCP values for the lungs, heart, and esophagus, whereas no significant differences were observed for the spinal cord.

**Conclusion:**

For SBBC patients requiring RNI, HFRT-VMAT achieved target coverage comparable to CFRT while reducing excessive high-dose regions and predicted toxicity to major thoracic organs. Despite slightly lower TCP values, overall tumor control remained favorable, supporting HFRT-VMAT as a feasible treatment option for this patient population.

## 1 Introduction

Radiotherapy poses a challenge for synchronous bilateral breast cancer (SBBC) with regional nodal irradiation (RNI) (1–4). Compared with unilateral disease, treatment involves irradiation of bilateral breast or chest wall targets in addition to the regional lymphatics, resulting in large and complex target volumes. This setup results in an increased dose to the heart and lungs and the trade-off between target coverage and normal tissue sparing becomes particularly challenging.

Hypofractionated radiotherapy (HFRT) is now widely used in the management of breast cancer with a strong radiobiological rationale and multiple randomized trials showing non-inferior tumour control with acceptable toxicity compared with conventional fractionation (5–7). However, most of this evidence is based on patients with unilateral disease and often lacks comprehensive RNI. In the case where SBBC requires RNI, the substantially larger target volume and increased planning complexity may affect both target dose coverage and radiation exposure to surrounding normal tissues, thereby potentially influencing tumor control and treatment-related toxicity (8). Therefore, dosimetric parameters alone may not be able to reflect the differences between hypofractionated and conventional fractionation regimens. Radiobiological assessment including modeling of TCP and NTCP may provide a more comprehensive assessment of the therapeutic ratio in this patient population. Given these considerations, the applicability of current hypofractionation data to SBBC patients needing RNI is uncertain and needs further investigation.

Against this clinical backdrop, volumetric modulated arc therapy (VMAT) is widely accepted because it can deliver highly conformal dose distributions with acceptable dose gradients to surrounding normal tissues (9). Recent technological advances have expanded the feasibility of bilateral breast irradiation. Novel VMAT-based approaches, including RapidArc Dynamic (10) and dynamic collimator rotation techniques (11), have shown potential to improve dose distribution and treatment efficiency while maintaining acceptable OAR sparing. However, the radiobiological implications of different fractionation schedules in SBBC patients requiring RNI remain insufficiently explored. Traditional plan evaluation based mainly on dose volume histogram (DVH) metrics are intrinsically descriptive and cannot be directly translated into clinically relevant endpoints such as tumor control or normal tissue toxicity. Radiobiological modeling offers a complementary mechanism for incorporating both the dose magnitude and the volume effects into quantitative outcome predictions. Using established models it is possible to estimate tumor control probability (TCP) and normal tissue complication probability (NTCP). These provide biologically interpretable indices in addition to purely physical dose parameters. This enables a more clinically relevant comparison of the competing treatment strategies, particularly in cases with heterogeneous dose distributions and competing trade-offs between the target coverage and the sparing of organs at risk (OARs).

The predictions of various radiobiological models are not consistent. TCP can be estimated by the Poisson-based linear quadratic (LQ-Poisson) model or Niemierko's equivalent uniform dose (EUD)-based model, while NTCP is typically calculated with the Lyman Kutcher Burman (LKB) model or EUD-based models. The consistency of these models has not been thoroughly investigated in the setting of SBBC with RNI.

In this study, we compare a hypofractionated VMAT regimen (40.05 Gy in 15 fractions) to a conventional regimen (50 Gy in 25 fractions) for SBBC with RNI. TCP are evaluated using LQ-Poisson and Niemierko models, NTCP using LKB and Niemierko models. This approach enables the comparison of fractionation strategies and the assessment of the consistency of radiobiological predictions, yielding a more robust basis for treatment selection in this challenging clinical setting.

## 2 Materials and methods

### 2.1 Patient selection and target delineation

Eighteen patients with histologically confirmed SBBC were retrospectively included in this study. All patients were scheduled to undergo comprehensive locoregional irradiation encompassing the bilateral breast and regional nodal volumes. CT simulation was performed for all patients in the supine position, with both arms elevated above the head to facilitate optimal target coverage and minimize beam obstruction. Immobilization was achieved using a dedicated wing board to ensure reproducible positioning throughout the treatment course. Patients were instructed to maintain free breathing during image acquisition. CT images were obtained with a slice thickness of 3 mm, providing adequate spatial resolution for accurate target delineation and OARs contouring.

Target volumes and OARs were delineated on the Eclipse treatment planning system (Varian Medical Systems, Palo Alto, CA) according to the Radiation Therapy Oncology Group (RTOG) contouring atlases for breast cancer. The clinical target volume (CTV) encompassed the bilateral whole breasts or chest wall together with the corresponding regional lymphatic nodal basins. Regional nodal volumes included the supraclavicular (level IV), axillary level III (infraclavicular region), and internal mammary chains, with additional inclusion of axillary nodal levels as clinically indicated based on the extent and distribution of disease. The planning target volume (PTV) was generated by applying a uniform 5 mm isotropic expansion to the CTV, followed by manual modification to exclude the superficial 3 mm beneath the skin surface. OARs delineated for each patient included the total lungs (right and left), heart, esophagus, and spinal cord.

### 2.2 Treatment planning

For each patient, two VMAT plans were generated: a HFRT regimen prescribing 40.05 Gy in 15 fractions and a conventionally fractionated radiotherapy (CFRT) regimen prescribing 50 Gy in 25 fractions. To ensure comparability, both plans were constructed using identical beam geometries. All plans employed a dual-isocenter technique, with isocenters placed at the geometric centers of the left and right breast targets, respectively. For each breast, two tangential partial arcs were designed to achieve adequate target coverage while reducing irradiation to adjacent OARs, particularly the heart and total lungs.

Treatment delivery was simulated using 6 MV flattened photon beams with a constant dose rate of 600 MU/min. The dose distribution was calculated with the Anisotropic Analytical Algorithm (AAA), using a spatial resolution of 2.5 mm for the calculation grid. Treatment planning was optimized to achieve full prescription coverage, with a minimum of 95% of the PTV encompassed by the prescribed dose level. Dose volume constraints for OARs were primarily derived from previously published dosimetric data in unilateral breast cancer, and were subsequently refined in consideration of emerging evidence from studies on SBBC, to enhance applicability to the present cohort while maintaining methodological consistency (12–16). These constraints were incorporated into the inverse optimization process. Iterative plan refinement was performed through progressive tightening of OAR dose objectives while maintaining adequate target coverage. Optimization was repeated until additional reductions in OAR doses resulted in unacceptable increases in dose heterogeneity or hot spot formation.

### 2.3 Dosimetric evaluation

Dosimetric evaluation of all treatment plans was performed using dose volume histogram (DVH) analysis. Target coverage and dose distribution within the PTV were assessed using V_95%_, V_107%_, and V_110%_, representing the percentages of the PTV receiving at least 95%, 107%, and 110% of the prescribed dose, respectively. Dose distribution characteristics within the target were further evaluated using D_2%_, D_50%_, and D_98%_, corresponding to the doses delivered to 2%, 50%, and 98% of the PTV volume, respectively.

The conformity index (CI) and homogeneity index (HI) were calculated to further evaluate dose conformity and dose uniformity within the target volume. A CI value approaching 1 indicated improved conformity of the prescribed dose to the target volume, whereas an HI value closer to 0 reflected a more homogeneous dose distribution within the target.

Target dose conformity was quantitatively assessed using the CI, calculated as follows (17):

$$\mathrm{CI}=TV_{\mathrm{PV}}^{2}/(\mathrm{TV}\cdot \mathrm{PV})$$

Where TV_PV_ denotes the portion of the target volume encompassed by the prescription isodose, TV corresponds to the total target volume, and PV represents the overall volume enclosed by the prescription isodose surface.

The HI was employed to quantify the degree of dose uniformity within the PTV, and is mathematically defined as (18):

$$\mathrm{HI}=(D_{2\text{\%}}-D_{98\text{\%}})/D_{50\text{\%}}$$

Where D_2%_ and D_98%_ correspond to the near-maximum and near-minimum doses within the target volume, respectively, and D_50%_ denotes the median dose.

### 2.4 Radiobiological modeling

Radiobiological effects of the different treatment regimens on both target volumes and OARs, including the total lungs, heart, esophagus, and spinal cord, were assessed using TCP and NTCP models based on DVH data derived from each treatment plan. TCP was calculated using the Niemierko and LQ-Poisson models, whereas NTCP was estimated using the Niemierko and LKB models.

#### 2.4.1 NTCP and TCP: Niemierko model

Physical dose distributions were initially converted into equivalent doses in 2 Gy fractions (EQD_2_) using the LQ model, allowing direct comparison between different fractionation regimens. The conversion was performed according to:

$$\mathrm{EQ}D_{2,i}=D_{i}\frac{\left(\alpha /\beta +d_{i}\right)}{\left(\alpha /\beta +2\right)}$$

Where D_i_ is the total physical dose delivered to the i-th dose-volume element, and d_i_ is the corresponding fractional dose for that volume element. The *α*/*β* ratio represents the tissue-specific parameter describing radiosensitivity within the framework of the linear-quadratic model.

The EUD was calculated according to the following expression to quantify the biological effect of non-uniform dose distributions (19, 20):

$$\mathrm{EUD}={\left(\sum_{i}v_{i}EQD_{2,i}^{a}\right)}^{\frac{1}{a}}$$

Where v_i_ represents the fractional volume of the structure receiving a dose D_i_, and *a* is a tissue-specific parameter that characterizes the volume-response effect of the organ or target under consideration.

The TCP and NTCP values were estimated using the following sigmoid dose–response function based on Niemierko's EUD model (20):

$$\mathrm{TCP}=\frac{1}{1+{\left(\frac{\mathrm{TC}D_{50}}{\mathrm{EUD}}\right)}^{4\cdot \gamma 50}}$$

$$\mathrm{NTCP}=\frac{1}{1+{\left(\frac{TD_{50}}{\mathrm{EUD}}\right)}^{4\cdot \gamma 50}}$$

Where TCD_50_ and TD_50_ denote the dose required to achieve a 50% probability of tumor control and normal tissue complication, respectively, while *γ*50 characterizes the steepness of the corresponding dose-response relationship at the 50% response level.

#### 2.4.2 TCP: LQ-Poisson model

TCP was calculated using a voxel-based Poisson model integrated with the LQ formalism and accounting for heterogeneous dose distribution. The analysis was based on differential DVH data, with physical doses converted to EQD_2_. The TCP was calculated according to the following expression (21, 22):

$$\mathrm{TCP}\;=\;\prod_{i=1}^{M}{\left[\exp\;\left(-\exp\left(e\cdot \;\gamma 50-\frac{\mathrm{EQ}D_{2,i}}{\mathrm{TC}D_{50}}\;\cdot (e\cdot \;\gamma 50-\ln(\ln2))\;\right)\;\right)\right]}^{vi}$$

Where M denotes the total number of dose bins in the differential DVH, v_i_ represents the fractional subvolume receiving the dose EQD_2, i_, and EQD_2, i_ corresponds to the equivalent dose in 2 Gy fractions for the i-th dose bin. The parameter TCD50 represents the dose associated with a 50% probability of response, while *γ*50 describes the normalized slope of the dose-response curve at the 50% response level.

#### 2.4.3 NTCP: LKB model

NTCP was estimated using the LKB radiobiological model. Within this model, NTCP is expressed as the upper tail of a standard normal distribution (23–25):

$$\mathrm{NTCP}=\frac{1}{\sqrt{2\pi }}\overset{t}{\underset{-\infty }{\int }}exp\left(-\frac{x^{2}}{2}\right)dx$$

$$t=\frac{D_{\mathrm{eff}}-TD_{50}}{m\cdot TD_{50}}$$

$$D_{\mathrm{eff}}={\left(\underset{i}{\sum }v_{i}{\mathrm{EQD}}_{2,i}^{\frac{1}{n}}\right)}^{n}$$

Where TD_50_ denotes the dose associated with a 50% probability of complication under uniform irradiation conditions, and m defines the slope of the dose-response curve at TD_50_. Here, D_i_ represents the dose delivered to the i-th dose bin, while v_i_ corresponds to the fractional volume receiving that dose. The parameter *n* is a tissue-specific constant describing the volume effect.

Model parameters were selected from previously published studies to maintain consistency with established radiobiological analyses in SBBC (4, 26, 27). Parameter selection was based on values most commonly adopted in comparable studies to ensure the reliability and reproducibility of the model calculations. A detailed summary of the radiobiological parameters used in the present analysis is provided in Table 1.

**Table 1:** Parameters used for the LQ-Poisson, LKB, and Niemierko models.

Organ	*α*/*β*	a	*γ*_50_	TCD_50_	TD_50_	*m*	*n*
PTV	4	−7.2	2	28	–	–	–
Heart	3	3	3	–	48	0.1	0.35
Lung	3	1	2	–	24.5	0.18	0.87
Spinal cord	2	13	4	–	66.5	0.175	0.05
Esophagus	3	19	4	–	68	0.11	0.06

PTV, planning target volume.

### 2.5 Statistical analysis

Continuous variables are presented as median and interquartile range (IQR). Comparisons between the HFRT and CFRT plans were performed using the Wilcoxon signed-rank test. A two-sided *P* value < 0.05 was considered statistically significant. Statistical analysis was performed using SPSS software (version 22.0, IBM Corp., Armonk, NY, USA).

## 3 Results

### 3.1 Dosimetric analysis for PTV

Both fractionation schemes achieved clinically acceptable target coverage for SBBC with RNI. Representative isodose distributions of CFRT and HFRT VMAT plans across the coronal, sagittal, and axial planes are presented in Figure 1. Dosimetric comparison demonstrated significant differences in several PTV parameters between the CFRT and HFRT plans (Table 2).

**Figure 1:**
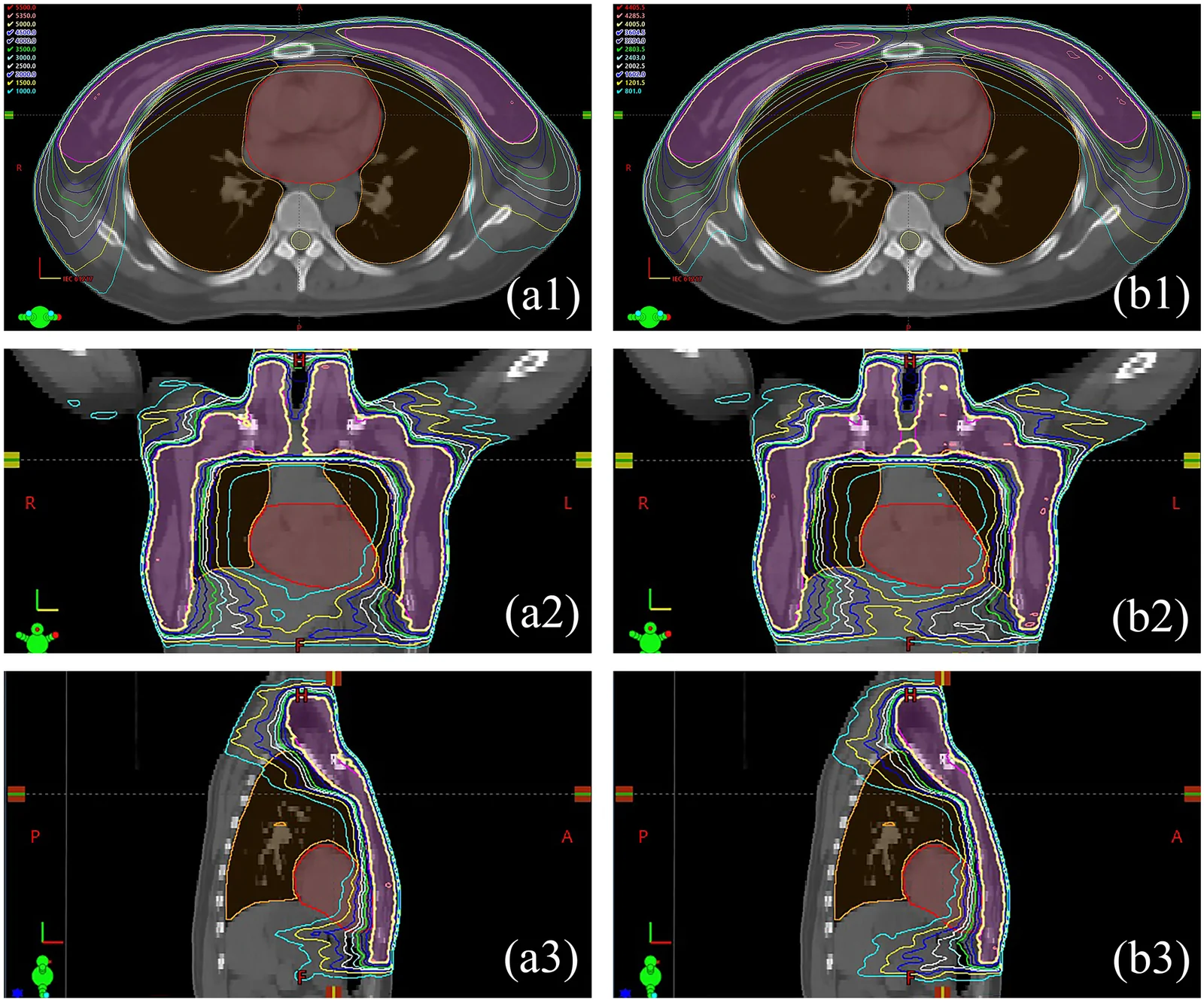
Representative coronal, sagittal, and axial views of the isodose distributions for CFRT **(a)** and HFRT **(b)** VMAT plans with RNI in patients with SBBC.

**Table 2:** Dosimetric parameters of the PTV in CFRT and HFRT plans.

Organ	Parameter	CFRT	HFRT	*z* Value	*P* Value
		Median (IQR)	Median (IQR)		
PTV	D_2%_(Gy)	53.46 (0.51)	42.99 (0.54)	−3.724	0.000
	D_98%_(Gy)	49.16 (0.26)	39.38 (0.23)	−3.726	0.000
	D_50%_(Gy)	52.11 (0.41)	41.79 (0.52)	−3.724	0.000
	V_95%_(%)	99.70 (0.23)	99.70 (0.20)	−1.387	0.166
	V_107%_(%)	2.60 (7.85)	1.35 (5.75)	−3.519	0.000
	V_110%_(%)	0.00 (0.00)	0.00 (0.00)	−1.342	0.180
	CI	0.83 (0.10)	0.82 (0.11)	−1.894	0.058
	HI	0.08 (0.01)	0.08 (0.02)	−1.809	0.065

PTV, planning target volume; CFRT, conventional fractionated radiotherapy; HFRT, hypofractionated radiotherapy; D_X%_(Gy), The doses received by X% of the target volume; V_X%_(%), The maximum volume receiving a specifc dose; CI, conformity index; HI, homogeneity index.

The HFRT plans showed lower absolute dose metrics because of the reduced prescription dose, with significantly lower D_2%_, D_98%_, and D_50%_ values compared with CFRT (all *P* < 0.01). Nevertheless, target coverage remained highly consistent between the two regimens, as reflected by comparable V_95%_ values exceeding 99% in both groups. No significant difference was observed in V_95%_ between the two regimens (*P* = 0.166).

Regarding dose homogeneity and conformity, both the CI and HI were comparable between the two approaches, with no statistically significant differences (*P* = 0.058 and *P* = 0.065, respectively). In addition, HFRT demonstrated lower high-dose exposure within the target volume, with significantly reduced V_107%_ compared with CFRT (CFRT 2.60% vs. HFRT 1.35%, *P* < 0.001), whereas no significant difference was identified for V_110%_ (*P* = 0.180). Overall, both VMAT strategies provided robust target coverage, while HFRT reduced excessive high-dose regions within the PTV.

### 3.2 Radiobiological evaluation of tumor control

Radiobiological analysis revealed significant differences in TCP between the two fractionation schedules (Table 3). The EUD of the PTV was significantly lower in HFRT than in CFRT (45.69 [0.48] Gy vs. 51.34 [0.34] Gy, *P* < 0.001).

**Table 3:** Comparison of radiobiological parameters between the CFRT and HFRT plans.

Organ	Parameter	CFRT	HFRT	*z* Value	*P* Value
		Median (IQR)	Median (IQR)		
PTV	EUD(Gy)	51.34 (0.34)	45.69 (0.48)	−3.725	0.000
	TCP_LQ Poisson(%)	95.82 (0.27)	90.60 (0.51)	−3.726	0.000
	TCP_Niemierko(%)	99.22 (0.03)	98.05 (0.16)	−3.728	0.000
Total Lungs	EUD(Gy)	9.84 (1.61)	9.46 (1.38)	−3.377	0.001
	NTCP_LKB(‰)	0.77 (0.76)	0.54 (0.55)	−3.680	0.000
	NTCP_Niemierko(‰)	0.68 (0.75)	0.49 (0.52)	−3.680	0.000
Heart	EUD(Gy)	12.39 (2.55)	11.06 (2.19)	−3.724	0.000
	NTCP_LKB(‰)	3.33 × 10^−11^ (1.47 × 10^−10^)	3.99 × 10^−12^ (1.23 × 10^−11^)	−3.724	0.000
	NTCP_Niemierko(‰)	8.73 × 10^−05^ (1.87 × 10^−04^)	2.24 × 10^−05^ (4.19 × 10^−05^)	−3.724	0.000
Spinal cord	EUD(Gy)	24.26 (4.02)	24.20 (2.96)	−1.416	0.157
	NTCP_LKB(‰)	0.31 (0.26)	0.27 (0.17)	−0.240	0.811
	NTCP_Niemierko(‰)	9.81 × 10^−05^ (1.78 × 10^−04^)	9.52 × 10^−05^ (1.41 × 10^−04^)	−0.152	0.879
Esophagus	EUD(Gy)	45.56 (1.64)	41.69 (1.59)	−3.726	0.000
	NTCP_LKB(‰)	1.04 (0.74)	0.17 (0.13)	−3.724	0.000
	NTCP_Niemierko(‰)	1.65 (0.89)	0.40 (0.23)	−3.724	0.000

PTV, planning target volume; CFRT, conventional fractionated radiotherapy; HFRT, hypofractionated radiotherapy; EUD, equivalent uniform dose; TCP, tumor control probability; NTCP, normal tissue complication probability; LQ, linear quadratic; LKB, Lyman Kutcher Burman.

Using the LQ-Poisson model, the median TCP values were 95.82 (0.27)% for CFRT and 90.60 (0.51)% for HFRT, demonstrating a statistically significant reduction in TCP with hypofractionation (*P* < 0.001). Similarly, the Niemierko model yielded significantly lower TCP values for HFRT (98.05 [0.16]%) compared with CFRT (99.22 [0.03]%, *P* < 0.001). Despite these statistical differences, TCP values remained high in both regimens, indicating satisfactory predicted tumor control for SBBC treated with VMAT and RNI.

### 3.3 Radiobiological evaluation of OARs

With respect to the total lungs, the HFRT plans significantly reduced pulmonary dose exposure compared with CFRT. The lung EUD decreased from 9.84 (1.61) Gy in CFRT to 9.46 (1.38) Gy in HFRT (*P* = 0.001). Correspondingly, both NTCP models demonstrated significantly lower predicted lung toxicity in HFRT. According to the LKB model, NTCP decreased from 0.77 (0.76)‰ to 0.54 (0.55)‰ (*P* < 0.001), while the Niemierko model showed a reduction from 0.68 (0.75)‰ to 0.49 (0.52)‰ (*P* < 0.001).

For the heart, HFRT also resulted in significantly lower cardiac dose exposure. The median heart EUD was reduced from 12.39 (2.55) Gy in CFRT to 11.06 (2.19) Gy in HFRT (*P* < 0.001). Extremely low cardiac NTCP values were observed for both fractionation schedules. Nevertheless, HFRT consistently demonstrated lower predicted cardiac complication probabilities than CFRT in both the LKB and Niemierko models (both *P* < 0.001).

Regarding the spinal cord, radiobiological parameters were comparable between the two fractionation schemes. No statistically significant differences were observed in EUD between CFRT and HFRT (24.26 [4.02] Gy vs. 24.20 [2.96] Gy, *P* = 0.157). Similarly, the NTCP values derived from both the LKB and Niemierko models showed no significant intergroup differences (*P* = 0.811 and *P* = 0.879, respectively).

The esophagus exhibited substantial reductions in dose exposure and predicted toxicity with HFRT. The esophageal EUD decreased significantly from 45.56 (1.64) Gy in CFRT to 41.69 (1.59) Gy in HFRT (*P* < 0.001). Consistently, the LKB model demonstrated a marked reduction in NTCP from 1.04 (0.74)‰ to 0.17 (0.13)‰ (*P* < 0.001), while the Niemierko model showed a decrease from 1.65 (0.89)‰ to 0.40 (0.23)‰ (*P* < 0.001). These findings suggest that HFRT may provide superior esophageal sparing during comprehensive nodal irradiation. Representative DVHs for the PTV, total lungs, heart, spinal cord, and esophagus are presented in Figure 2, providing a visual comparison of dose distributions between CFRT and HFRT.

**Figure 2:**
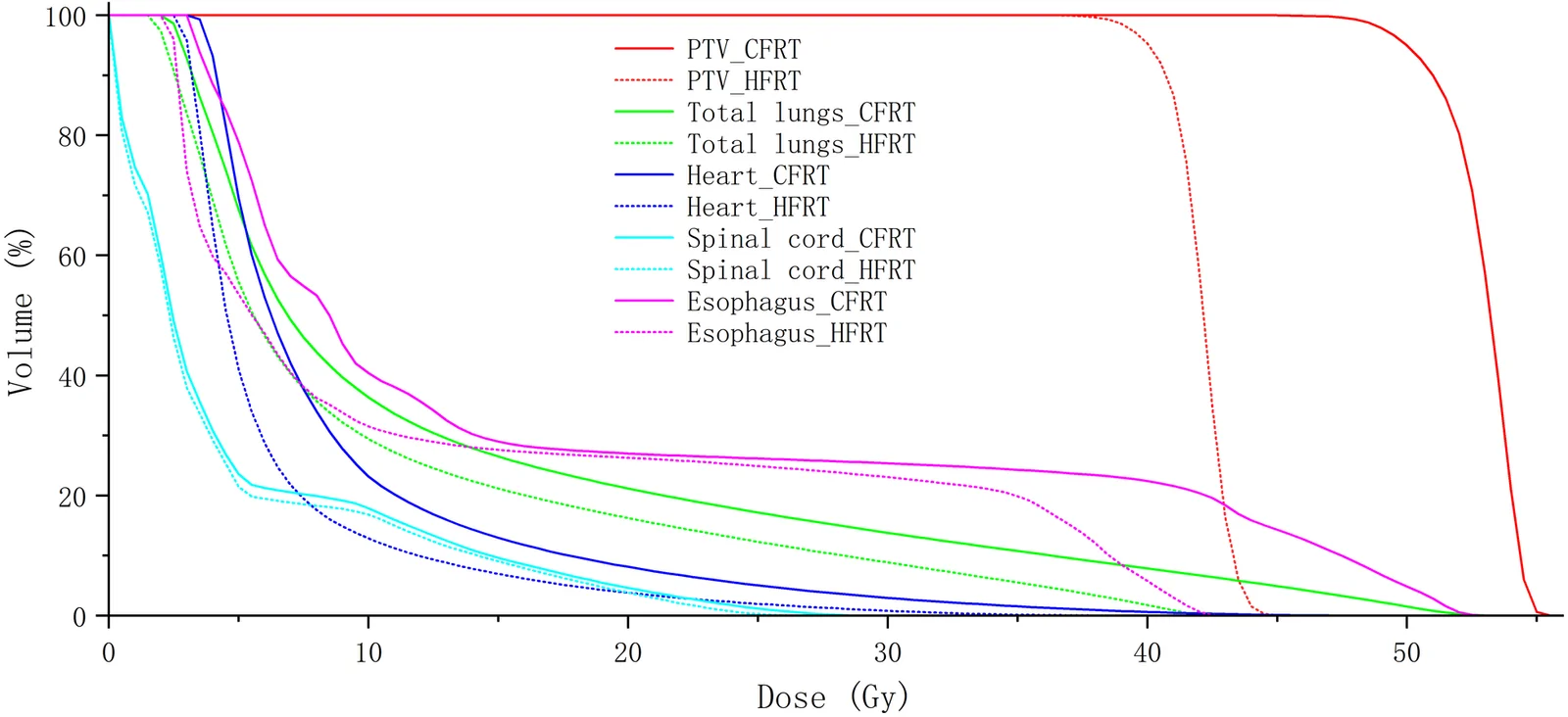
Representative DVHs for CFRT and HFRT VMAT plans in a patient with SBBC receiving RNI. DVHs are shown for the PTV, total lungs, heart, spinal cord, and esophagus.

## 4 Discussion

The present study compared HFRT and CFRT using VMAT for SBBC with RNI, with particular emphasis on radiobiological response. The findings demonstrated that both fractionation schemes achieved excellent target coverage and clinically acceptable dose distributions. Although HFRT showed slightly lower TCP values than CFRT in both the LQ-Poisson and Niemierko models, substantial reductions in predicted normal tissue toxicity were observed for the lungs, heart, and esophagus. These results suggest that hypofractionated VMAT may provide a favorable therapeutic balance in SBBC patients requiring comprehensive nodal treatment. To our knowledge, this is one of the first studies to specifically report the safety, feasibility, tolerability, and radiobiological implications of a 3-week hypofractionated regimen (40.05 Gy in 15 fractions) for SBBC patients, including those requiring RNI. Gadea et al. previously demonstrated that this schedule is associated with minimal acute toxicity and excellent early disease control in a mixed cohort of SBBC patients, with no grade ≥3 acute side effects and a 95% disease-free status at a median follow-up of 9 months (14). These findings indicate that radiobiological evaluation provides complementary information beyond conventional dosimetric assessment by simultaneously considering tumor control and normal tissue toxicity. For instance, Arjmand et al. also reported that while conventional regimens achieved higher TCP, hypofractionated regimens (particularly 39 Gy/13 fractions) led to significantly lower NTCP for the lungs and heart compared to conventional 50 Gy/25 fractions (21).

SBBC represents a technically demanding clinical scenario because of the large and complex target volume, overlap between bilateral tangential fields, and the close proximity of critical thoracic organs. The incorporation of RNI further increases treatment complexity by enlarging the irradiated volume and elevating the risk of cardiopulmonary exposure. VMAT has emerged as an effective technique for SBBC owing to its ability to improve conformity and dose homogeneity while simultaneously sparing surrounding OARs (1). A systematic review by Protopapa et al. concluded that VMAT can achieve high-quality treatment plans for SBBC when appropriate planning parameters, including isocenter selection and beam arrangement, are adopted (28). Our use of a dual-isocenter VMAT technique with multiple partial arcs aligns with previously reported strategies aimed at reducing the low-dose bath to the lungs while maintaining adequate target coverage. In the present study, both CFRT and HFRT plans achieved excellent PTV coverage, with V95% values exceeding 99% in both groups. Moreover, the conformity indices were comparable, indicating that dose conformity was maintained despite the lower prescription dose in HFRT. Our findings are consistent with previous reports demonstrating that VMAT can achieve excellent target conformity and high predicted tumor control in SBBC (26).

The HFRT plans demonstrated significantly lower V107% values, indicating a reduction in excessive high-dose regions within the target. This finding is clinically relevant because dose hotspots in bilateral breast irradiation may contribute to acute skin toxicity, fibrosis, and compromised cosmetic outcomes (29). No significant difference in HI was observed between the two fractionation schedules, suggesting that dose homogeneity was well maintained with HFRT despite the lower prescription dose. Previous dosimetric studies of bilateral breast VMAT have similarly reported that hypofractionated schedules can maintain acceptable target homogeneity while reducing unnecessary dose spill to adjacent tissues (30). These findings indicate that HFRT can reduce excessive high-dose regions without compromising overall dose homogeneity.

Radiobiological evaluation revealed lower TCP values for HFRT compared with CFRT using both models. This result is likely attributable to the lower total prescription dose delivered in the hypofractionated regimen despite the larger dose per fraction. However, the absolute TCP values remained high, particularly in the Niemierko model, where TCP exceeded 98% in the HFRT group. This finding is consistent with several published reports. Similarly, Kazemzadeh et al. reported EUD-based TCP values of 99.16% for CFRT and 95.96% for HFRT (40 Gy/15 fractions), demonstrating the same trend as observed in the present study (31). Tai et al., using the Halcyon system, reported that although the CFRT plan achieved a slightly higher TCP than the HFRT plan (95.5% vs. 93.6%), the HFRT regimen yielded a greater biologically effective dose relative to the prescribed dose, with the EUD corresponding to 114% and 107% of the prescription dose for HFRT and CFRT, respectively (22). Similarly, in the present study, the HFRT plan demonstrated a higher relative EUD compared with the CFRT plan (114% vs. 103% of the total prescribed dose), further supporting the enhanced biological dose intensity achievable with hypofractionated VMAT. These findings suggest that hypofractionation regimens may provide favorable biological dose escalation while maintaining clinically acceptable target coverage and normal tissue sparing, although further optimization and long-term clinical validation remain necessary. Large randomized trials in unilateral breast cancer, including START-A, START-B, and the Canadian hypofractionation trial, have shown equivalent local control between hypofractionated and conventional regimens (5–7). Although SBBC remains less extensively studied, the present results suggest that the radiobiological effectiveness of HFRT may also extend to bilateral disease treated with advanced VMAT techniques.

An important finding of this study was the consistent reduction in predicted normal tissue complications with HFRT. Pulmonary toxicity is a major concern in SBBC because bilateral irradiation inevitably increases lung exposure. In the present analysis, HFRT significantly reduced lung EUD and yielded lower NTCP values in both radiobiological models. Even though the absolute NTCP values remained low in both regimens, the relative reduction observed with HFRT may still be clinically meaningful, particularly in patients with preexisting pulmonary disease or those receiving systemic therapies associated with pulmonary toxicity. Reduced treatment fractions may additionally decrease the cumulative inflammatory burden to lung tissue. This advantage of HFRT for lung sparing is supported by the findings of Arjmand et al., who noted that the 39 Gy/13 fraction regimen showed the lowest lung NTCP (21), and Tai et al., who reported lower NTCP for the ipsilateral lung with HFRT plans (3.7% vs. 4.9% for CFRT) (22). However, it is worth noting that this is not a universal finding; Kazemzadeh et al. paradoxically reported a higher lung NTCP for HFRT (0.13) compared to CFRT (0.024), highlighting that outcomes can be highly dependent on the specific radiobiological parameters and treatment planning techniques used (31). Furthermore, Wu et al. demonstrated that optimization of beam arrangement can further reduce lung dose during VMAT treatment (3).

Cardiac sparing remains a critical issue in breast radiotherapy, especially in bilateral treatment combined with RNI. Epidemiological studies have shown that even small increases in mean heart dose may elevate the long-term risk of ischemic heart disease (32). In the present study, HFRT significantly lowered cardiac EUD and demonstrated consistently reduced NTCP estimates compared with CFRT. This finding is strongly supported by Patil et al., who reported that in SBBC, HFRT (40.05 Gy/15 fractions) reduced mean heart dose from 5.53 Gy (CFRT) to 4.59 Gy, with a corresponding decrease in cardiac mortality NTCP (Poisson-LQ model) from 0.30% to 0.16% (30). Although the absolute NTCP values were extremely low, the observed reduction supports the potential long-term benefit of hypofractionation when combined with optimized VMAT planning. This cardiac-sparing effect of HFRT is a robust finding across multiple studies. Consistent with our findings, both Arjmand et al. and Kazemzadeh et al. reported heart NTCP values of zero for HFRT (21, 31), while Zhou et al. reported that VMAT techniques further reduced cardiac dose levels under HFRT (27). These findings suggest that HFRT, when integrated with modern VMAT techniques, can achieve favorable cardiac sparing without compromising target coverage, thereby supporting its clinical feasibility in the management of SBBC.

The esophagus exhibited the most pronounced reduction in predicted toxicity among the evaluated OARs. This finding likely reflects the reduced integral dose associated with the shorter hypofractionated regimen. Esophageal toxicity is particularly relevant in patients receiving supraclavicular and internal mammary nodal irradiation, as the esophagus is often exposed to low and intermediate radiation doses. The markedly lower NTCP values observed with HFRT may translate into improved treatment tolerance and reduced acute swallowing discomfort during therapy. Balasubramanian and Shobana also evaluated esophageal dose in SBBC, noting that while mean doses were similar across techniques, the LKB model predicted a higher NTCP for HTRT plans compared to IMRT and VMAT, underscoring the importance of technique selection in esophageal sparing (26). Our results, showing a significant reduction in esophageal EUD and NTCP with HFRT, suggest that hypofractionation may further mitigate this toxicity, a hypothesis that warrants prospective validation.

In contrast, no significant differences were observed for spinal cord EUD or NTCP. This result suggests that spinal cord dose was already effectively constrained during optimization in both fractionation schedules and remained well below clinically relevant tolerance thresholds. The consistently low spinal cord NTCP values further confirm the safety of VMAT-based bilateral breast irradiation when appropriate planning constraints are applied. This finding is consistent with Balasubramanian and Shobana, who reported negligible NTCP values for the spinal cord across all studied techniques (26).

The current study highlights the value of integrating radiobiological modeling into dosimetric evaluation. Conventional DVH parameters provide important physical dose information but may not fully reflect biological response differences between fractionation schedules. By incorporating TCP and NTCP analyses, the present work offers a more comprehensive assessment of the therapeutic ratio between HFRT and CFRT in SBBC. These findings demonstrate that radiobiological modeling provides clinically meaningful information beyond conventional dosimetric evaluation. Although dosimetric differences between HFRT and CFRT were relatively modest, consistent reductions in predicted NTCP together with maintained high TCP suggest that radiobiological evaluation may facilitate treatment selection for SBBC patients receiving VMAT with RNI. The combined use of LQ-Poisson, Niemierko, and LKB models also improved the robustness of the analysis by reducing dependence on a single predictive approach. However, our results also highlight the significant inter-model variability reported in the literature. Specifically, while the LQ-Poisson model yielded lower TCP estimates, the Niemierko model predicted TCP values exceeding 98% for both treatment plans, a phenomenon also observed by Balasubramanian and Shobana (26). This underscores the fact that these models are highly sensitive to input parameters (e.g., *α*/*β*, TCD50, *γ*50), and absolute values should be interpreted with caution, primarily using them for relative comparisons between treatment plans.

Several limitations should be acknowledged. First, this was a dosimetric and modeling-based study without clinical outcome validation. TCP and NTCP models are influenced by parameter selection and may not perfectly predict actual patient outcomes. Nevertheless, Gadea et al. provided clinical corroboration for the safety of the 40.05 Gy/15 fractions schedule in SBBC, reporting no grade ≥3 acute toxicity and a 95% disease-free rate at early follow-up, which supports the clinical relevance of our modeling predictions (14). Second, this was a single-center study with a relatively limited sample size. This reflects the rarity of SBBC, particularly among patients requiring RNI. Therefore, the generalizability of the present findings should be interpreted with caution. Third, respiratory motion remains a significant source of uncertainty, particularly in free-breathing SBBC treatments. While we did not employ motion management, Zhou et al. have demonstrated the feasibility and benefit of using an Optical Surface Monitoring System to manage intrafraction motion, and future studies on SBBC should consider incorporating such technologies, especially with deep inspiration breath hold (DIBH), to further reduce cardiopulmonary doses (27). Relatedly, DIBH was not evaluated in the present study. DIBH is an established heart-sparing approach, particularly for left-sided breast or chest wall irradiation with regional nodal involvement. Our retrospective analysis was based on free-breathing CT datasets, and corresponding DIBH datasets were not available for a consistent paired comparison. Therefore, the dosimetric and radiobiological findings should not be extrapolated to DIBH-based treatment or interpreted as evidence that free-breathing VMAT provides equivalent cardiac protection. Future studies should evaluate the combined effects of fractionation, VMAT geometry, and DIBH in patients with SBBC requiring RNI. Future studies with larger patient cohorts and prospective toxicity assessment are warranted. In addition, long-term clinical follow-up is necessary to determine whether the radiobiological advantages observed with HFRT translate into measurable reductions in late toxicity and equivalent tumor control in SBBC patients.

## 5 Conclusion

Hypofractionated VMAT for SBBC with RNI achieved excellent target coverage and acceptable dose homogeneity while significantly reducing predicted toxicity to major thoracic organs compared with conventional fractionation. Although slightly lower TCP values were observed with HFRT, tumor control remained high across both radiobiological models. These findings support the feasibility and potential clinical benefit of hypofractionated VMAT as an effective treatment strategy for SBBC requiring comprehensive nodal irradiation.

## Data Availability

The raw data supporting the conclusions of this article will be made available by the authors, without undue reservation.
